# Association of early and mid-pregnancy maternal serum uric acid with hypertensive disorders of pregnancy

**DOI:** 10.3389/fendo.2025.1731576

**Published:** 2025-12-12

**Authors:** Chang Zou, Ruru Zhao, Xiaosong Liu, Yuanyuan Yang, Qinxin Shen, Qiaoling Du

**Affiliations:** Shanghai Key Laboratory of Maternal Fetal Medicine, Shanghai Institute of Maternal-Fetal Medicine and Gynecologic Oncology, Shanghai First Maternity and Infant Hospital, School of Medicine, Tongji University, Shanghai, China

**Keywords:** uric acid, pregnancy-induced hypertension, preeclampsia, eclampsia, gestational hypertension (GH)

## Abstract

**Objective:**

To examine association between maternal serum uric acid (UA) measured during gestational 4–24 weeks and the subsequent development of hypertensive disorders of pregnancy (HDP) in a large Chinese cohort.

**Methods:**

This historical cohort study included 84,298 singleton pregnancies registered at Shanghai First Maternity and Infant Hospital (2013–2022). Serum UA was measured before 24 weeks of gestation. Participants with pre-existing hypertension or incomplete data were excluded. Generalized additive models (GAMs) and multivariable logistic regression analyses were used to assess nonlinear and independent associations between UA levels (quartiles and continuous values) and risks of gestational hypertension (GH), preeclampsia (PE), and overall HDP cases, adjusting for maternal age, pre-pregnancy BMI, education, and glucose metabolism disorders.

**Results:**

UA levels were consistently higher among women who later developed HDP than in normotensive pregnancies throughout gestational weeks 4–24. Higher UA concentrations consistently associated with an increased risk of HDP diseases, the top UA quartile showed the strongest associations with GH (1.82, 1.59–2.08), PE (1.67, 1.48–1.89), and total HDP (1.77, 1.61–1.94). GAM analyses revealed enhanced relation of UA to HDP occurrence from the 4^th^ week to 24^th^ week, and showed specific patterns in GH/PE, predictive strength of maternal UA increased with advancing gestational age.

**Conclusions:**

Elevated maternal UA levels in early-to-mid gestation independently related to HDP risk, with subtype-specific and gestational-age–dependent patterns. UA serves as a practical potential biomarker for early risk stratification and dynamic monitoring of women at risk for hypertensive complications.

## 1 Introduction

Hypertensive disorders of pregnancy (HDP), encompassing gestational hypertension (GH), preeclampsia and eclampsia (PE), represent a major global health burden, persistently ranking among the top causes of maternal and perinatal morbidity and mortality (1, 2). Accounting for approximately 10% of pregnancy-related complications (3, 4), HDP contributes significantly to adverse outcomes such as preterm birth, intrauterine growth restriction, placental abruption (5), and long-term cardiovascular disease risk in affected mothers (6). Despite considerable progress in prenatal care and obstetric management (7), the underlying pathophysiology of HDP remains incompletely elucidated, with current evidence pointing to a complex interplay of placental dysfunction, endothelial injury, systemic inflammation, and oxidative stress (8). It partially hinders the development of reliable early prediction tools, leaving clinicians to rely on late-appearing clinical signs such as hypertension and proteinuria, markers that often emerge only after irreversible placental damage has occurred. Consequently, the identification of features in populations with high HDP risk has become a critical priority in maternal-fetal medicine.

Among the array of hypertensive disease biomarkers, serum uric acid (UA) has garnered increasing attention (9) due to its pathophysiological relevance in HDP. UA is the end product of purine metabolism (10), an active contributor to endothelial dysfunction, oxidative stress, and renal impairment (11), and the key mechanistic pathways implicated in HDP development. Elevated serum UA levels have been consistently associated with increased risk of hypertensive diseases across diverse populations (12, 13), pregnant women and HDP are also included (14, 15), and the hyperuricemia in this context is thought to arise from multiple mechanisms, including reduced renal excretion secondary to vasoconstriction and glomerular endotheliosis, heightened xanthine oxidase activity (16) driven by placental ischemia-reperfusion injury, and increased cellular turnover due to oxidative stress (17). UA concentrations were significantly higher in women progressing to severe preeclampsia or eclampsia compared to those without severe features (18). Though there has been research on the relation between UA and HDP, few of them provide a large-scale cohort of clinical cases to support their conclusion; the same problem also exists in such studies on the Chinese population. Moreover, when being used as a standalone predictor, the effectiveness of UA can be influenced by confounding variables such as maternal hydration status and pre-pregnancy metabolic conditions, which need further adjustment in the analysis procedures.

This study aims to investigate the relation between maternal serum UA measured during early and mid-pregnancy (before 24^th^ weeks’ gestation) and the subsequent developed HDP, specifically based on Chinese pregnant women. We provide longitudinal data extracted from a large, well-characterized prospective cohort to test the hypothesis that elevated UA in early- to mid-pregnancy is associated with high risk of HDP, independent of other typical maternal risk factors. By employing rigorous statistical methods, we seek to provide robust evidence to guide clinical decisions.

## 2 Methods

### 2.1 Participant enrollment and ethics approval

**Figure 1:**
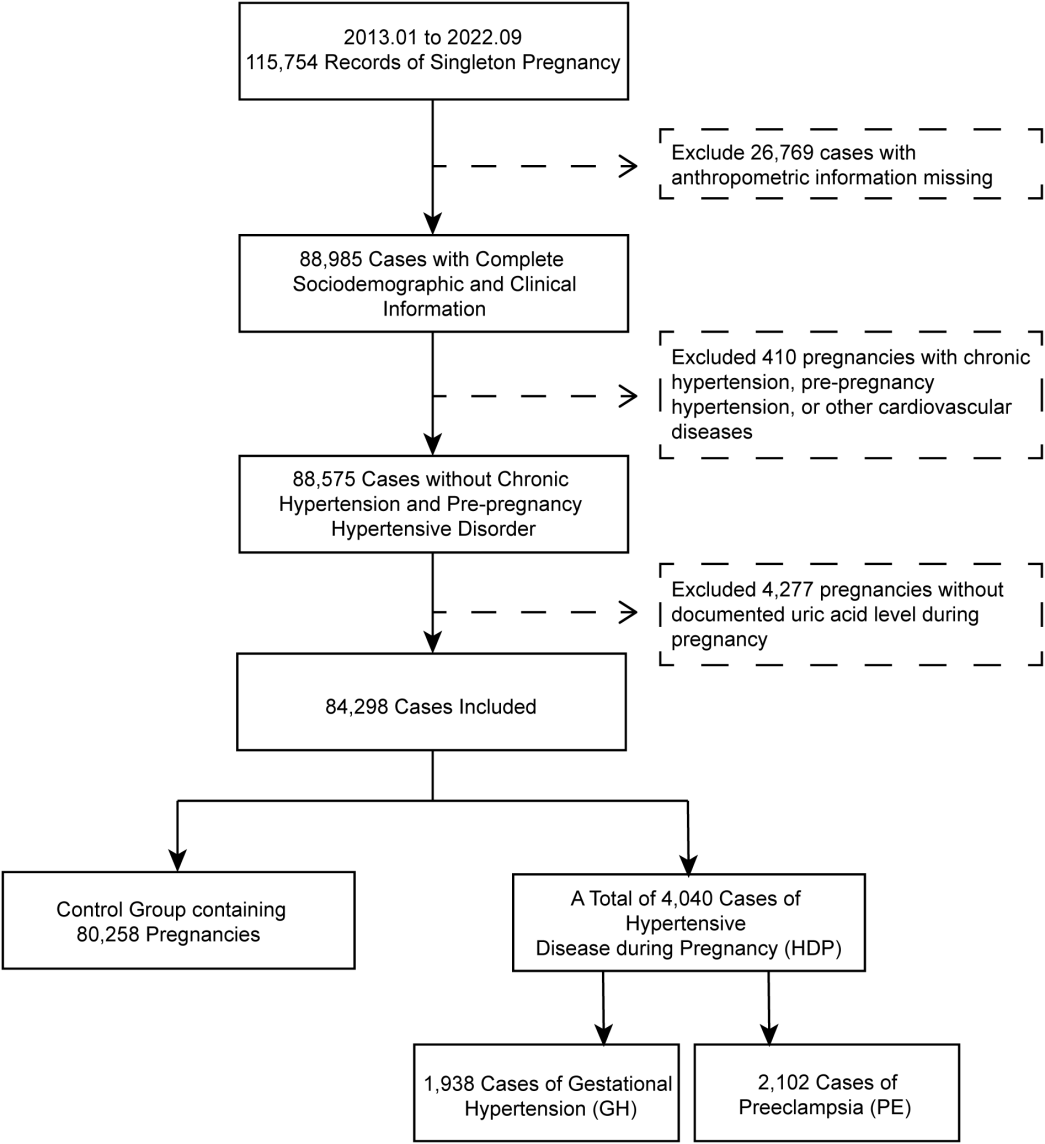
Demographic and clinical characteristics of study population by HDP outcome.

This historical cohort research was conducted at Shanghai First Maternity and Infant Hospital in China. We analyzed singleton pregnancies in women registered between January 2013 and September 2022. Exclusion criteria included: 1) Pre-existing hypertension or other cardiovascular diseases; 2) Implausible gestational age or incomplete clinical information and UA data records; 3) duplicate pregnancy records. Finally, 84,298 eligible cases were enrolled (**Figure 1**). Demographic and clinical data were prospectively collected through standardized clinical documentation, including age at conception, residential origin (divided into Shanghai/non-Shanghai), education level of the mother, pre-pregnancy anthropometrics (height, weight), and the calculated body mass index (BMI, weight[kg]/height[m]²). Gestational age was calculated from the last menstrual period. The diagnoses of “gestational hypertension”, “preeclampsia”, and “eclampsia”, as well as the mode of delivery, were retrieved from the electronic records. This study has received approval from the Institutional Review Board (IRB) of the Shanghai First Maternity and Infant Hospital, School of Medicine, Tongji University (Ethics Approval Number: KS1998). Abbreviations mentioned in this work were listed in **Supplementary Table S1**.

### 2.2 Serum UA measurement

Serum UA levels were measured during every prenatal examination before the 24^th^ week of gestation. Fasting venous blood samples were collected at the clinic, and immediately centrifuged at 4,500 rpm for serum extraction. Levels of serum UA were quantified using enzymatic methods on a Hitachi 7600 chemical analyzer (Hitachi Co., Tokyo, Japan) at the clinical laboratory of our hospital. The intra-assay coefficient of variation (CV) in the clinical laboratory was below 4.25% and the inter-assay CV was below 5.67%. All measurements were performed in the same hospital laboratory using a consistent analytic platform with daily internal quality-control procedures and annual external proficiency testing throughout the study period. Though temporal assay drift or batch effects across the 10-year period cannot be entirely ruled out, such bias would likely be non-differential.

### 2.3 Statistical analysis

All statistical analyses were performed using R software (version 4.3.0). Continuous variables were presented as mean ± standard deviation or median (interquartile range) as appropriate, while categorical variables were expressed as counts (percentages). For the longitudinal analysis, we used Kendall’s rank correlation test to evaluate trends in UA levels over time within each group. The median and interquartile ranges of UA levels were calculated for each gestational window (the 4^th^-8^th^, 9^th^-12^th^, 13^th^-16^th^, 17^th^-20^th^, and 21^st^-24^th^ weeks).

Potential nonlinear relationships were explored by generalized additive models (GAMs) with logit link functions with a cubic regression spline and a basis dimension of k = 10 for gestational weeks, and internal validation was performed by 10-fold cross-validation, to accommodate potential nonlinear relationships and interaction effects between UA and other linear confounders. In this way, the nonlinear associations between serum UA levels and the risk of each HDP outcome were revealed, respectively; separate models were fitted with smooth terms and interaction terms. Adjusted predictions were generated to visualize the probability of HDP outcomes across UA levels at representative gestational weeks. The non-faceted plots were derived via locally weighted scatterplot smoothing (LOESS); the faceted plots were estimated using GAMs. The above analyses were conducted in R using the mgcv package for GAMs.

Multivariable logistic regression models were also developed to assess the independent association between UA levels and HDP risk after adjusting for potential confounders. As an exposure variable, UA levels were categorized into quartiles (Q1-Q4). The models adjusted for: 1) maternal age (per 5-year increase); 2) pre-pregnancy BMI; 3) glucose metabolism disorder (GMD); 4) maternal education level. Adjusted odds ratios (aORs) with 95% confidence intervals were calculated for each variable. Tests for linear trend across UA quartiles were performed by modeling quartiles as an ordinal variable. Model assumptions were verified using variance inflation factors (all <2.0) and Hosmer-Lemeshow goodness-of-fit tests (all p>0.05).

All statistical tests were two-sided, with p-values <0.05 considered statistically significant. Data visualization was performed using the ggplot2 package, including smoothed curves for GAM predictions and forest plots for odds ratios. About missing data handling, for each analysis, pregnancies with missing exposure or outcome information would be excluded. Given the small amount of missing data and lack of systematic patterns in missingness, such exclusion hardly introduced meaningful bias in the estimated associations.

## 3 Results

### 3.1 Profiles of the study population divided by HDP development

The study included a total of 84,298 pregnancies (**Figure 1**), with their demographic and clinical information exhibited based on the diagnosis of hypertensive diseases (**Table 1**). Maternal age distribution across grouped pregnancy cases seems to be similar, while subjects with HDP diagnosis had significantly higher pre-pregnancy BMI (GH: 23.2 ± 3.7; PE: 22.9 ± 3.5; HDP: 23.1 ± 3.7) compared to normotensive pregnancies (21.2 ± 2.7), Moreover, the prevalence of pre-pregnancy obesity was significantly higher in GH (9.9%), PE (7.5%), and HDP (8.6%) compared to normotensive women (2.2%), meanwhile women with PE (17.1%) and HDP (16.2%) had higher rates of glucose metabolism disorders than normotensive women (11.2%), including pre-gestational diabetes mellitus (PGDM) and impaired glucose tolerance (IGT). Majority of the enrolled pregnant women held bachelor’s degree, while lower proportions of bachelor (Normotensive 61.0% vs PE 56.6%, HDP 58.8%) and advanced education (Normotensive 14.2% vs GH 9.4%, PE 12.1%, HDP 10.8%), and higher proportions of low education (Normotensive 8.0% vs GH 9.5%, PE 9.9%, HDP 9.8%) were observed in cases with hypertensive diseases.

**Table 1:** Demographic and clinical profiles of study population by development of HDP.

	Normotensive (N = 80258)	GH (N = 1938)	PE (N = 2102)	HDP (N = 4040)
Demographic Characteristics				
Age (years)	30.2 ± 3.7	30.5 ± 4.1	30.3 ± 4.0	30.4 ± 4.0
Pre-pregnancy BMI (kg/m²)	21.2 ± 2.7	23.2 ± 3.7	22.9 ± 3.5	23.1 ± 3.7
Residential Origin (Shanghai, %)	17529 (21.8%)	598 (30.9%)	547 (26.0%)	1145 (28.3%)
Education Level				
Bachelor's Degree	48981 (61.0%)	1186 (61.2%)	1190 (56.6%)	2376 (58.8%)
Master's/Doctoral Degree	11409 (14.2%)	182 (9.4%)	254 (12.1%)	436 (10.8%)
Associate Degree	13464 (16.8%)	385 (19.9%)	449 (21.4%)	834 (20.6%)
High School or Less	6404 (8.0%)	185 (9.5%)	209 (9.9%)	394 (9.8%)
Clinical Characteristics				
Gestational Obesity (N%)	1767 (2.2%)	191 (9.9%)	158 (7.5%)	349 (8.6%)
Glucose Metabolism Disorder (N%)	8971 (11.2%)	294 (15.2%)	359 (17.1%)	653 (16.2%)
Uric Acid Levels				
Uric Acid (μmol/L)	200 (174, 229)	219 (190, 255)	218 (186, 251)	219 (188, 253)

The table contrasts 84,298 singleton pregnancies grouped by hypertensive outcome: normotensive control (n = 80,258), gestational hypertension (GH, n = 1,938), preeclampsia (PE, n = 2,102) and all hypertensive disorders of pregnancy (HDP, n = 4,040). Values are mean ± SD, median (P25, P75) or N (%). BMI, Body Mass Index; GH, Gestational Hypertension; PE, Preeclampsia; HDP, Hypertensive Disorder during Pregnancy.

### 3.2 Distribution of maternal serum UA across gestational age

Remarkably, the average level of UA across each case’s all gestational age was elevated in GH (190–255μmol/L), PE (186–251μmol/L), and combined HDP cohorts (188–253μmol/L) compared to normotensive women (174–229μmol/L) (**Table 1**).

**Figure 2:**
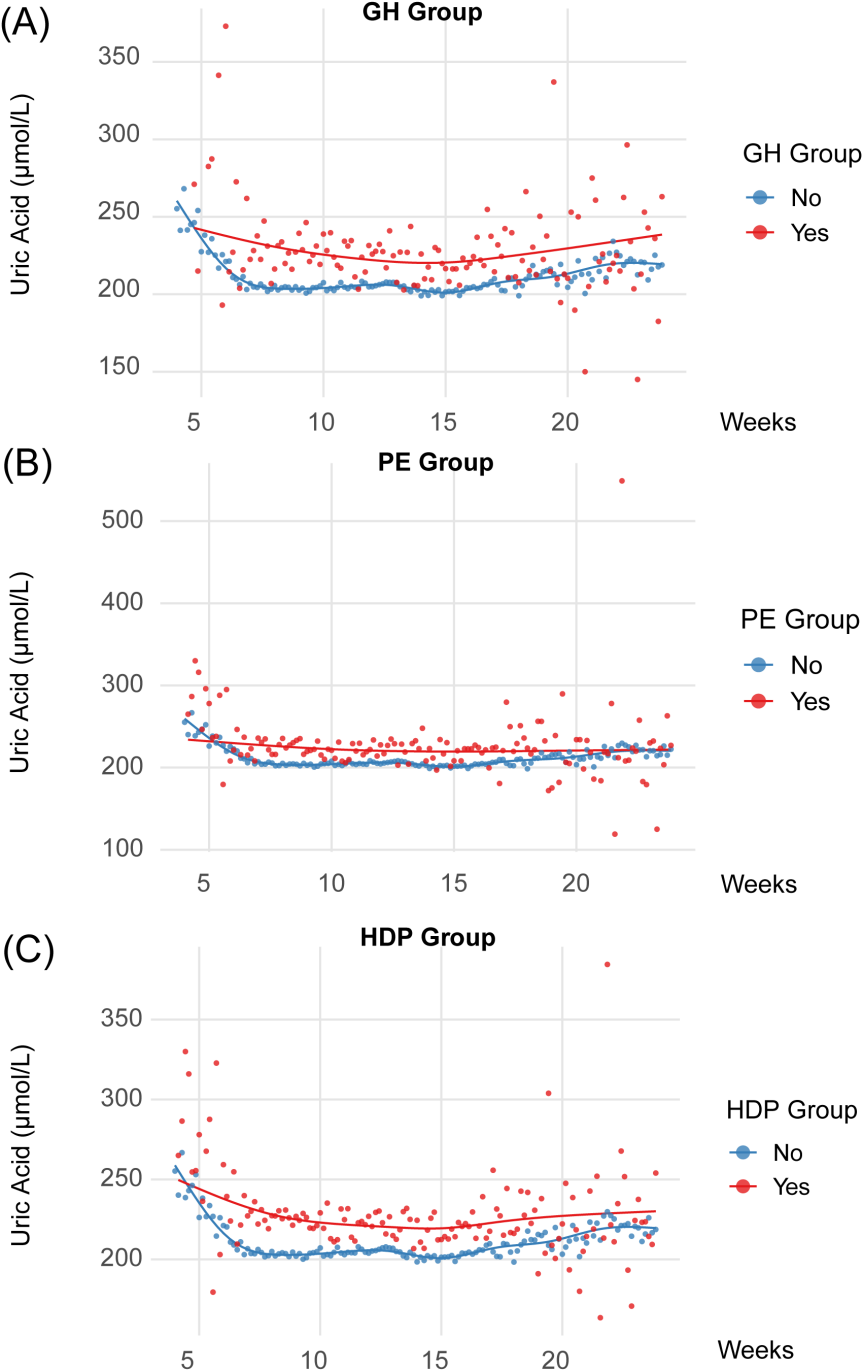
Trends of uric acid (UA) levels across gestational weeks stratified by pregnancy outcomes. This figure illustrates the trajectories of UA concentrations (mmol/L) over gestational weeks for three hypertensive disorders of pregnancy. **(A)** Comparison between gestational hypertension (GH) group and normotensive group; **(B)** Comparison between preeclampsia (PE) group and normotensive group; **(C)** Comparison between overall hypertensive disorders (HDP) group and normotensive group. Each panel displays smoothed trends (with 95% confidence bands) using GAM for cases (red) and non-cases (blue), with points representing mean UA values at each gestational week.

Across the 4^th^-8^th^ week and the afterward every 4-week gestational window until the 24^th^ week, UA levels were consistently higher in women who later developed HDP (including GH and PE) than in normotensive ones (**Table 2**), and such consistent elevation was also illustrated in **Figure 2**. From the gestation of 4^th^–24^th^ weeks, pregnancies later diagnosed with HDP exhibited persistently higher UA levels than normotensive pregnancies, with no discernible temporal trend across the whole gestational age, which means serum UA changed significantly with advancing gestational age, yet no consistent or interpretable pattern emerged across these changes.

**Table 2:** Longitudinal serum UA variations in early to mid-pregnancy.

	Counts	Uric Acid (μmol/L)
Control		
*4–8 weeks*	8,125	202 (175, 233)
*9–12 weeks*	30,325	199 (173, 229)
*13–16 weeks*	31,460	200 (174, 227)
*17–20 weeks*	12,138	203 (178, 230)
*21–24 weeks*	1,688	211 (182, 244)
Gestational hypertension		
*4–8 weeks*	191	221 (192, 272)
*9–12 weeks*	738	220 (191, 257)
*13–16 weeks*	765	215 (185, 247)
*17–20 weeks*	312	224 (192, 255)
*21–24 weeks*	55	234 (208, 270)
Preeclampsia		
*4–8 weeks*	236	225 (192, 259)
*9–12 weeks*	954	217 (185, 250)
*13–16 weeks*	744	218 (187, 249)
*17–20 weeks*	273	214 (186, 250)
*21–24 weeks*	60	220 (174, 256)
Hypertensive disorder during pregnancy		
*4–8 weeks*	427	224 (192, 265)
*9–12 weeks*	1,692	218 (188, 254)
*13–16 weeks*	1,509	216 (186, 248)
*17–20 weeks*	585	220 (189, 253)
*21–24 weeks*	115	226 (194, 263)

The table presents maternal serum uric acid concentrations [μmol/L, median (interquartile range)] measured at the gestations of 4–8, 9–12, 13–16, 17–20 and 21–24 weeks, stratified by hypertensive outcome: control (n = 83,736 measurements), gestational hypertension (n = 2,061), preeclampsia (n = 2,267), and overall hypertensive disorders of pregnancy (n = 4,328). Repeated measurements per woman within the same pregnancy were allowed in the dataset.

### 3.3 GAMs reveal the association between maternal UA levels across early- to mid-gestation and HDP outcomes

In our GAMs models using log-transformed maternal uric acid [log(UA)] (**Supplementary Figure S1**) as a continuous predictor, the associations between UA levels and the risk of HDP diseases varied across gestational weeks, with distinct temporal patterns to different subtypes of HDP.

**Figure 3:**
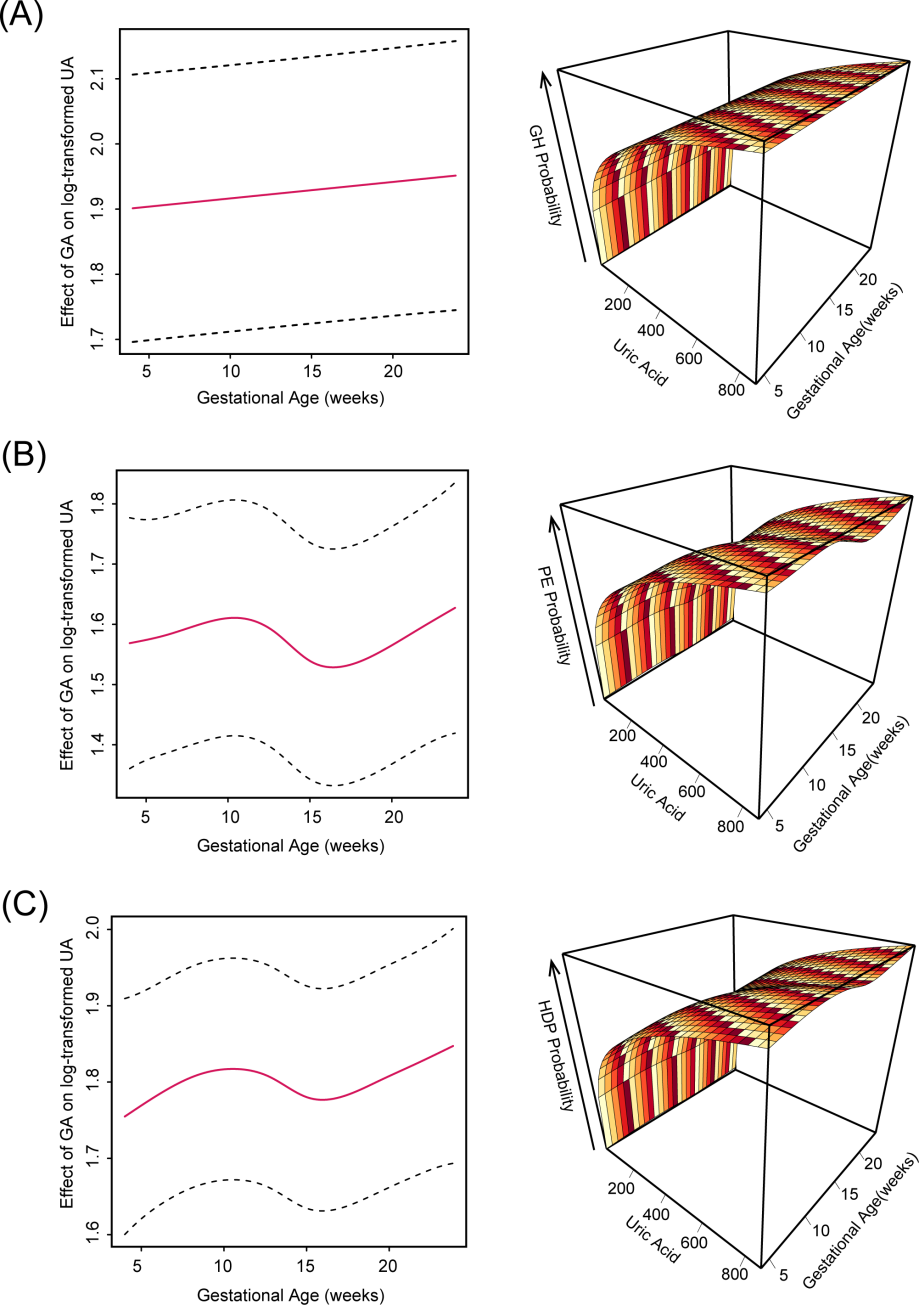
Interaction effects of UA on hypertensive outcomes via GAM. **(A)** Interaction effect of UA on GH outcome; **(B)** Interaction effect of UA on PE outcome; **(C)** Interaction effect of UA on the overall HDP outcome. Marginal nonlinear effects of UA on log-odds of each outcome are exhibited. The 3D interaction plots reveal complex, nonlinear associations where UA effects vary significantly by gestational age, particularly after mid-pregnancy.

**Figure 4:**
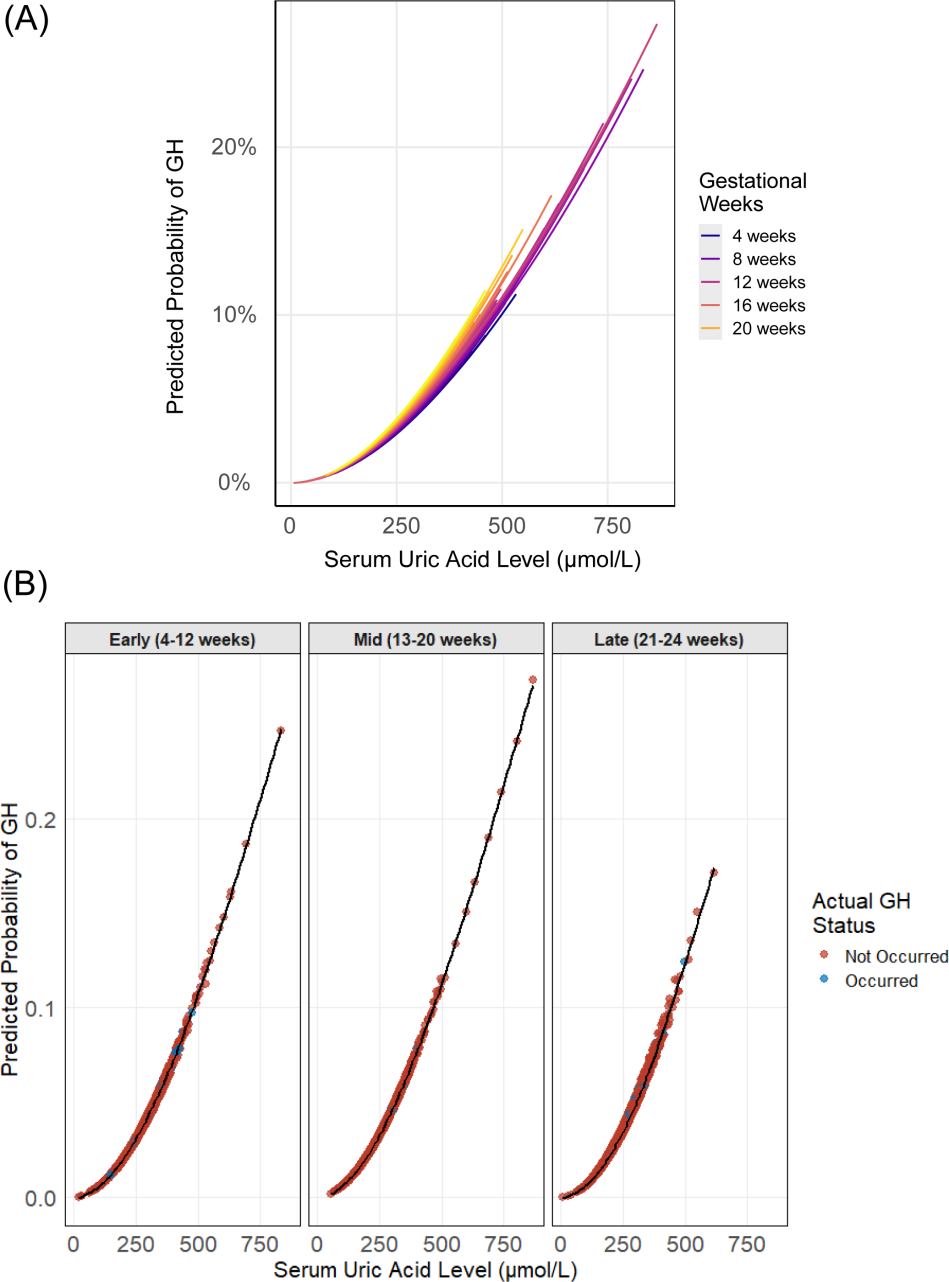
Predicted probabilities of GH by UA levels and gestational age. **(A)** Continuous UA-probability relationships colored by gestational weeks (4–24 weeks), with loess-smoothed trends (shaded 95% CI). **(B)** UA effects across three gestational periods (Early 4–12 weeks; Mid 13–20 weeks; Late 21–24 weeks) using GAM-smoothed curves, with point colors representing outcome status.

**Figure 5:**
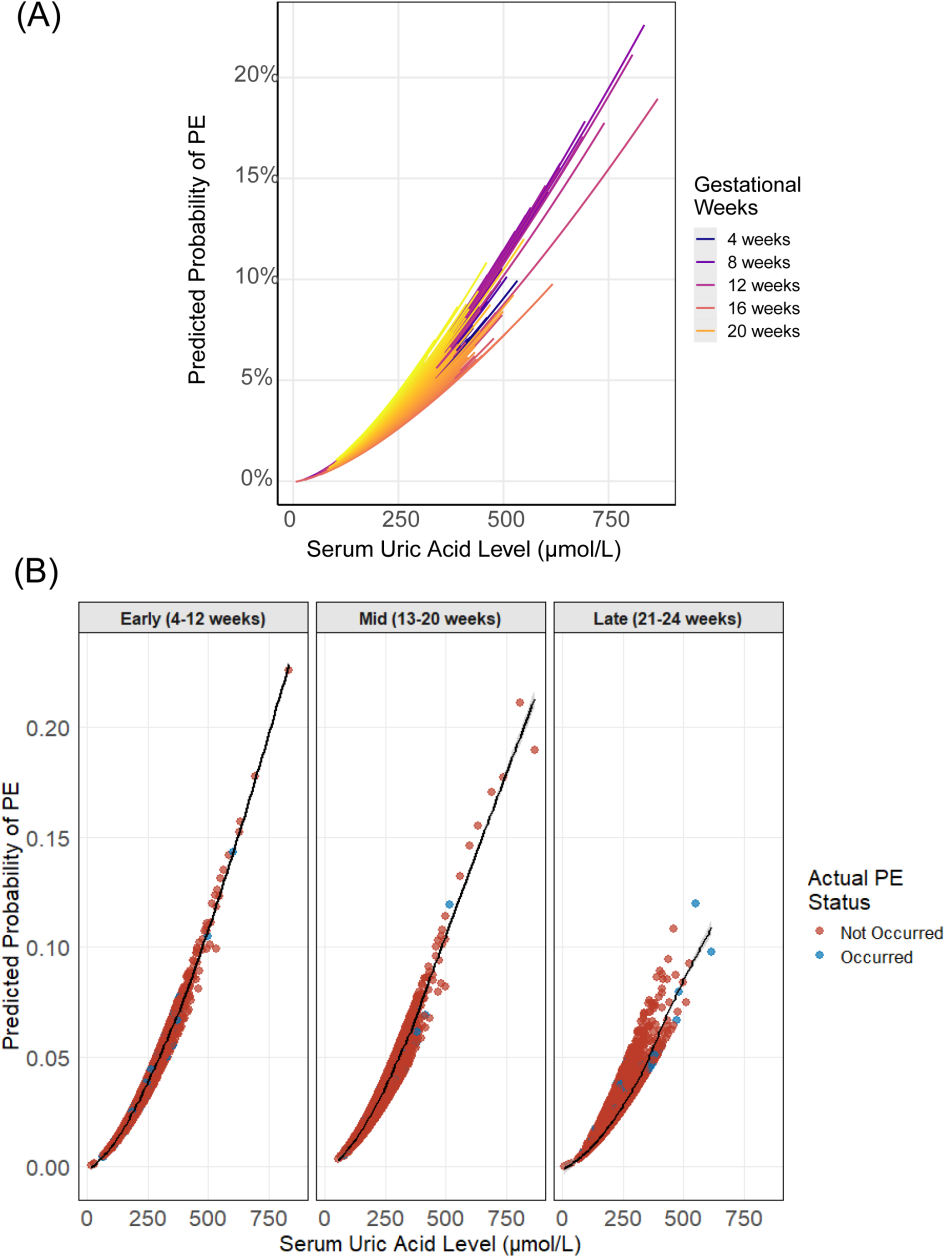
Predicted probabilities of PE by UA levels and gestational age. **(A)** Continuous UA-probability relationships colored by gestational weeks (4–24 weeks), with loess-smoothed trends (shaded 95% CI). **(B)** UA effects across three gestational periods (Early 4–12 weeks; Mid 13–20 weeks; Late 21–24 weeks) using GAM-smoothed curves, with point colors representing outcome status.

**Figure 6:**
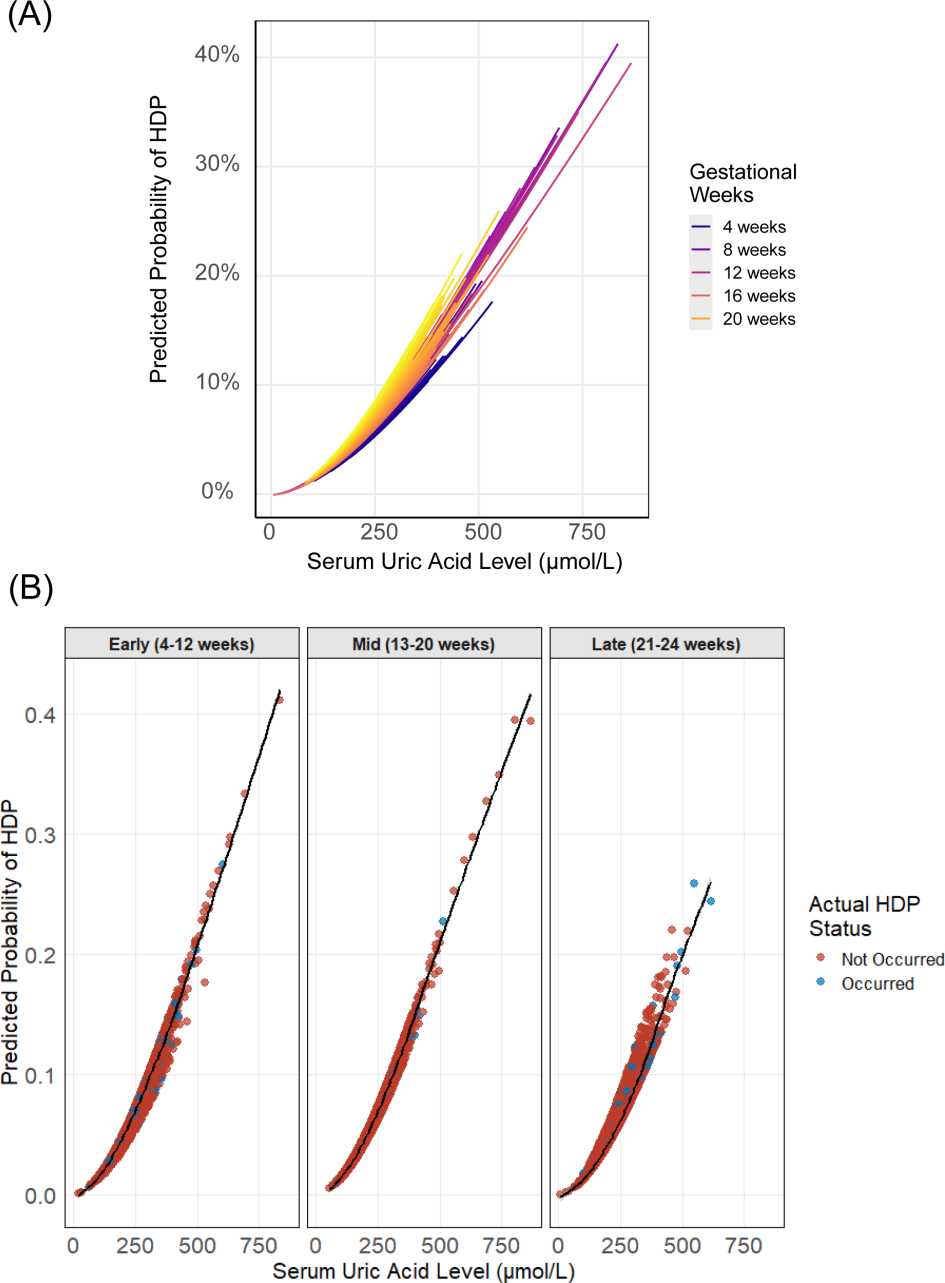
Predicted probabilities of overall HDP by UA levels and gestational age. **(A)** Continuous UA-probability relationships colored by gestational weeks (4–24 weeks), with loess-smoothed trends (shaded 95% CI). **(B)** UA effects across three gestational periods (Early 4–12 weeks; Mid 13–20 weeks; Late 21–24 weeks) using GAM-smoothed curves, with point colors representing outcome status.

Overall, higher maternal UA concentrations were consistently associated with an increased risk of HDP, at any gestational week between 4^th^ to 24^th^ weeks and for all HDP subtypes (**Figures 3**–**6**). For GH (**Figure 3A**), the estimated association of log(UA) with adverse outcome was approximately linear, indicating a stable positive relationship throughout 4^th^ to 24^th^ gestational weeks [effective degrees of freedom (edf) = 2.00, χ² = 357.7, p < 0.0001]. As for PE (**Figure 3B**), the relationship displayed a non-linear N-shaped curve, with two peaks of association strength occurring around gestational weeks 10 and 24 (edf = 5.60, χ² = 333.1, p < 0.0001). In the GAM analysis simultaneously including GH and PE cases (**Figure 3C**), the smooth term of gestational week interacting with maternal log(UA) was highly significant (edf = 5.51, χ² = 662.3, p < 0.0001). It indicated a markedly nonlinear association between log(UA) and HDP risk over gestation. The fitted smooth function showed a pattern resembling the GAM model of PE, characterized by an overall N-shaped relationship with more pronounced upward trends in later gestational weeks.

Furthermore, LOESS curves representing the predicted risks of the adverse outcomes based on the established GAM models were shown. It’s obvious that GH showed the most stable enhanced relation between maternal UA and hypertensive outcome from 4^th^ to 24^th^ weeks’ gestation (**Figure 4**) among all the models. Though for both PE and overall HDP models, associations between maternal UA levels and outcomes varied non-monotonically across gestational weeks with great significance, higher UA concentrations were still associated with an increased risk of HDP diseases (**Figures 4**, **5**).

### 3.4 Association of maternal UA level with HDP risks adjusted by maternal covariates

Supported by previous analyses, elevated UA within 4^th^-24^th^ weeks may signify a sustained pro-hypertensive state. The research further conducted multivariable regression analyses using UA as the exposure variable and adjusted maternal covariates. It revealed significant associations between elevated UA and increased risks of GH, PE, and any HDP after confounder adjustments (**Table 3**). Compared to the lowest UA quartile (Q1), women with the highest quartile (Q4) had substantially higher odds of GH (aOR=1.82, 95%CI: 1.59–2.08), PE (aOR=1.67, 95%CI: 1.48–1.89), and the combined risk of HDP (aOR=1.77, 95%CI: 1.61–1.94)(all p<0.001), with intermediate risk for Q3 (GH aOR=1.41, 95%CI: 1.23-1.63; PE aOR=1.17, 95%CI: 1.02-1.33; HDP aOR=1.28, 95%CI: 1.16–1.41) and no significant association for Q2, indicating evident dose-response relationship.

**Table 3:** Multivariable-adjusted associations of UA quartiles with HDP risk.

	*GH OR (95% CI)*	*PE OR (95% CI)*	*HDP OR (95% CI)*
Uric Acid Quartiles			
*Q1 (Reference)*	1.00 (ref)	1.00 (ref)	1.00 (ref)
*Q2*	1.08 (0.93-1.25)	0.99 (0.87-1.14)	1.03 (0.93-1.14)
*Q3*	1.41 (1.23-1.63) ^***^	1.17 (1.02-1.33) ^*^	1.28 (1.16-1.41) ^***^
*Q4*	1.82 (1.59-2.08) ^***^	1.67 (1.48-1.89) ^***^	1.77 (1.61-1.94) ^***^
Age (per 5-year increase)	1.11 (1.05-1.18) ^***^	1.00 (0.95-1.06)	1.05 (1.01-1.09) ^*^
Pre-pregnancy BMI	1.18 (1.16-1.19) ^***^	1.15 (1.13-1.16) ^***^	1.17 (1.16-1.18) ^***^
GMD	0.97 (0.85-1.09)	1.21 (1.08-1.36) ^***^	1.09 (1.00-1.19) ^*^
Education Level			
*Bachelor (Reference)*	1.00 (ref)	1.00 (ref)	1.00 (ref)
*Master's/Doctoral Degree*	0.71 (0.61-0.82) ^***^	1.03 (0.90-1.17)	0.87 (0.78-0.96) ^**^
*Associate Degree*	1.10 (0.98-1.23)	1.28 (1.15-1.42) ^***^	1.20 (1.11-1.30) ^***^
*High School or Less*	1.02 (0.87-1.19)	1.26 (1.09-1.45) ^**^	1.15 (1.03-1.28) ^*^

Adjusted odds ratios (aOR) and 95% confidence intervals (CI) for GH, PE, and any HDP, stratified by UA quartiles and adjusted for maternal age, pre-pregnancy BMI, glucose metabolism disorder (GMD), and education level. The reference group for UA was the lowest quartile (Q1). Significance levels: ***p<0.001, **p<0.01, *p<0.05. BMI, Body Mass Index; CI, Confidence Intervals; GH, Gestational Hypertension; GMD, Glucose Metabolism Disorder; OR, Odds Ratios; PE, Preeclampsia; HDP, Hypertensive Disorder during Pregnancy.

Moreover, pre-pregnancy BMI showed a strong effect, with each unit increase associated with 16–19% higher odds of GH, 13–16% higher odds of PE, and totaling 16–18% higher odds of HDP (all p<0.001); and each 5-year more advanced conception age linked to higher 11% GH risk (p<0.001). GMDs were associated with PE risk (aOR=1.21, p<0.001). Risks of the three hypertensive outcomes were also differentially adjusted by maternal education level: advanced education above bachelor’s degree served as protective factor against GH (aOR=0.71, p<0.001) and any HDP development (aOR=0.87, p<0.01), whereas associate education or lower associated with increased PE risk (aOR=1.28 for associate degree, p<0.001; aOR=1.26 for high school education or less, p<0.01), as well as the whole HDP risk (aOR=1.20 for associate degree, p<0.001; aOR=1.15 for high school education or less, p<0.05).

**Figure 7:**
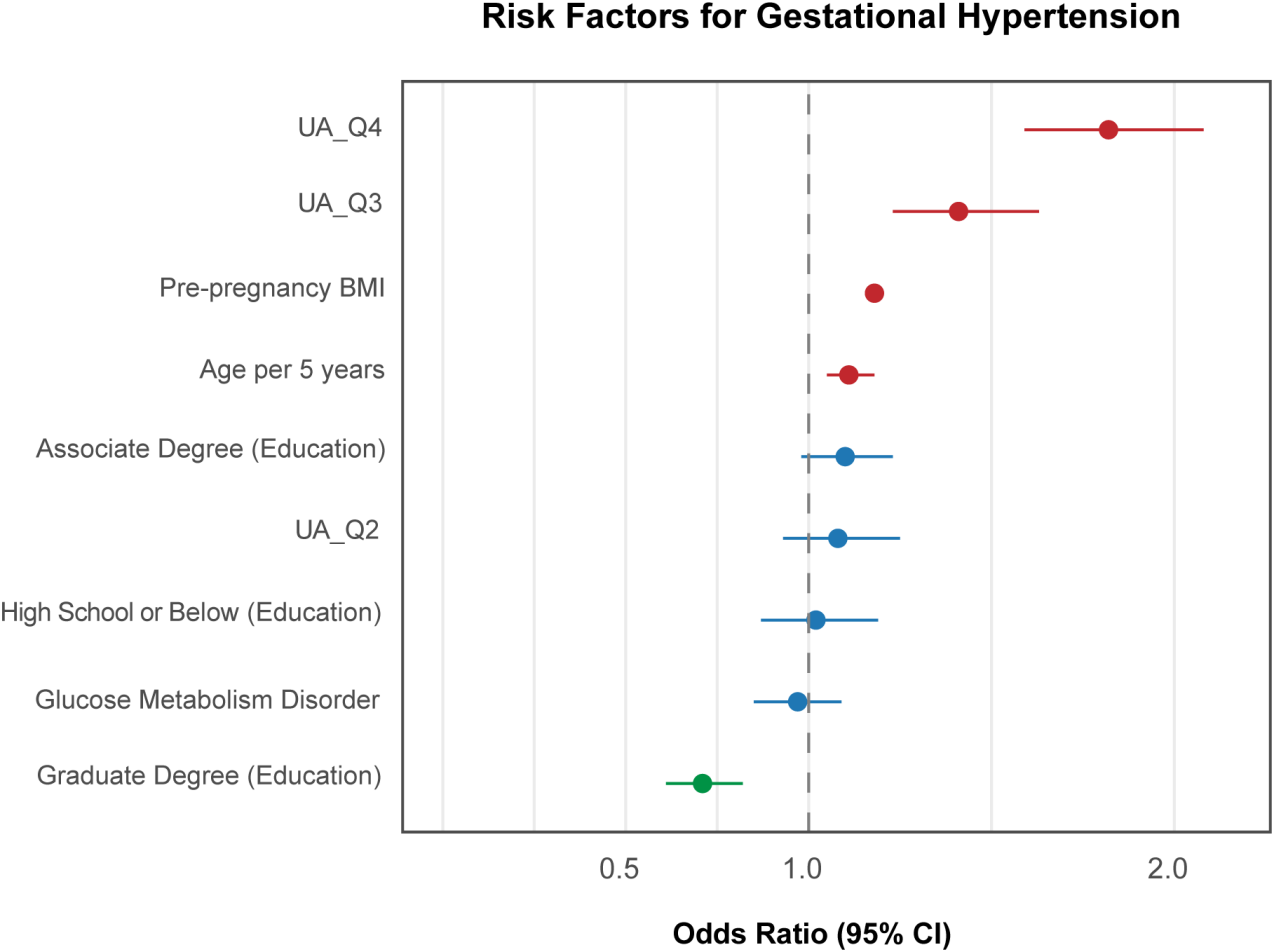
Forest plot of adjusted odds ratios (aOR) for GH. This forest plot shows the relationship between UA quartiles and GH, adjusting for education, parity, BMI, diabetes mellitus, and age per 5-year increase.

**Figure 8:**
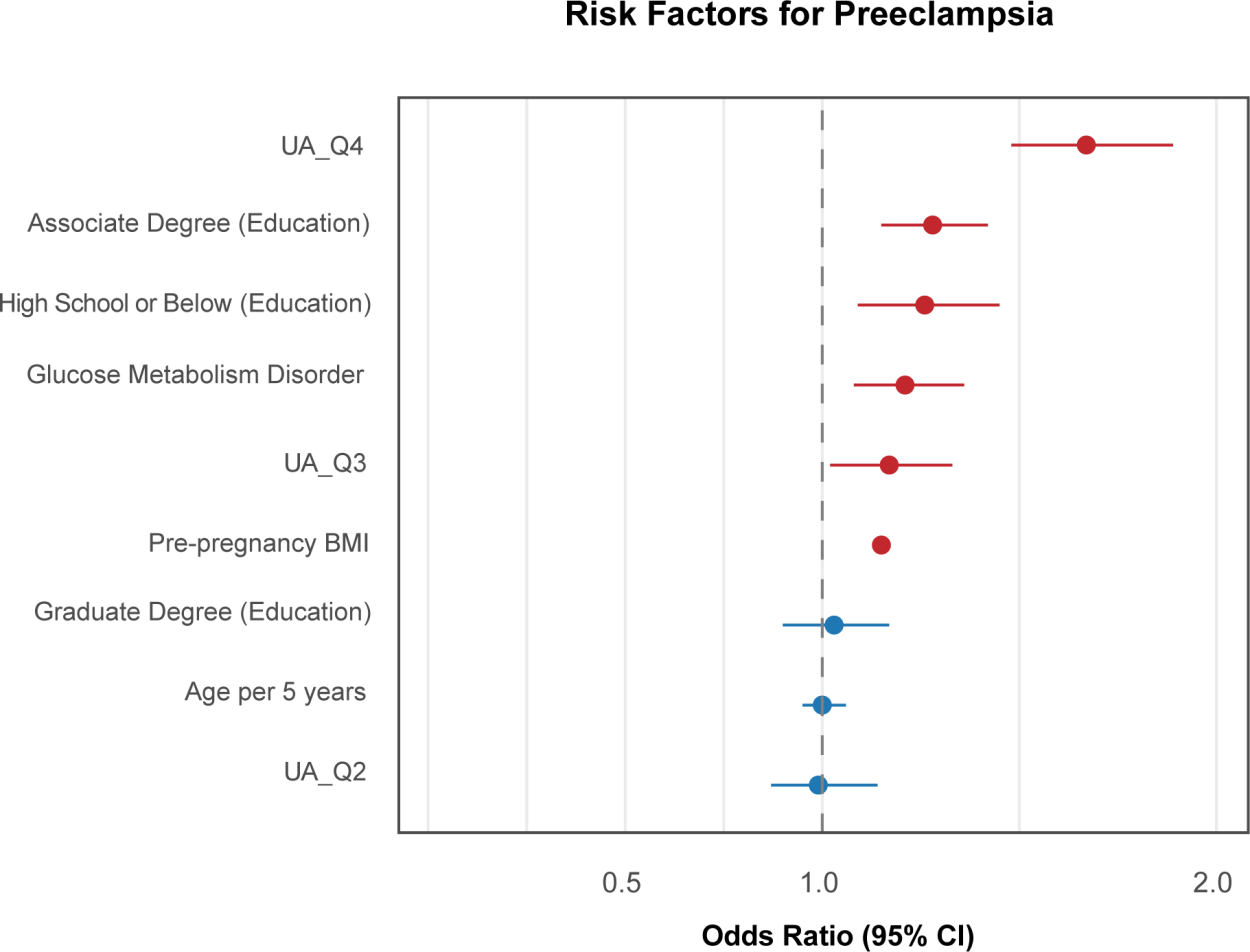
Forest plot of aOR for PE. This forest plot shows the relationship between UA quartiles and PE, adjusting for education, parity, BMI, diabetes mellitus, and age per 5-year increase.

**Figure 9:**
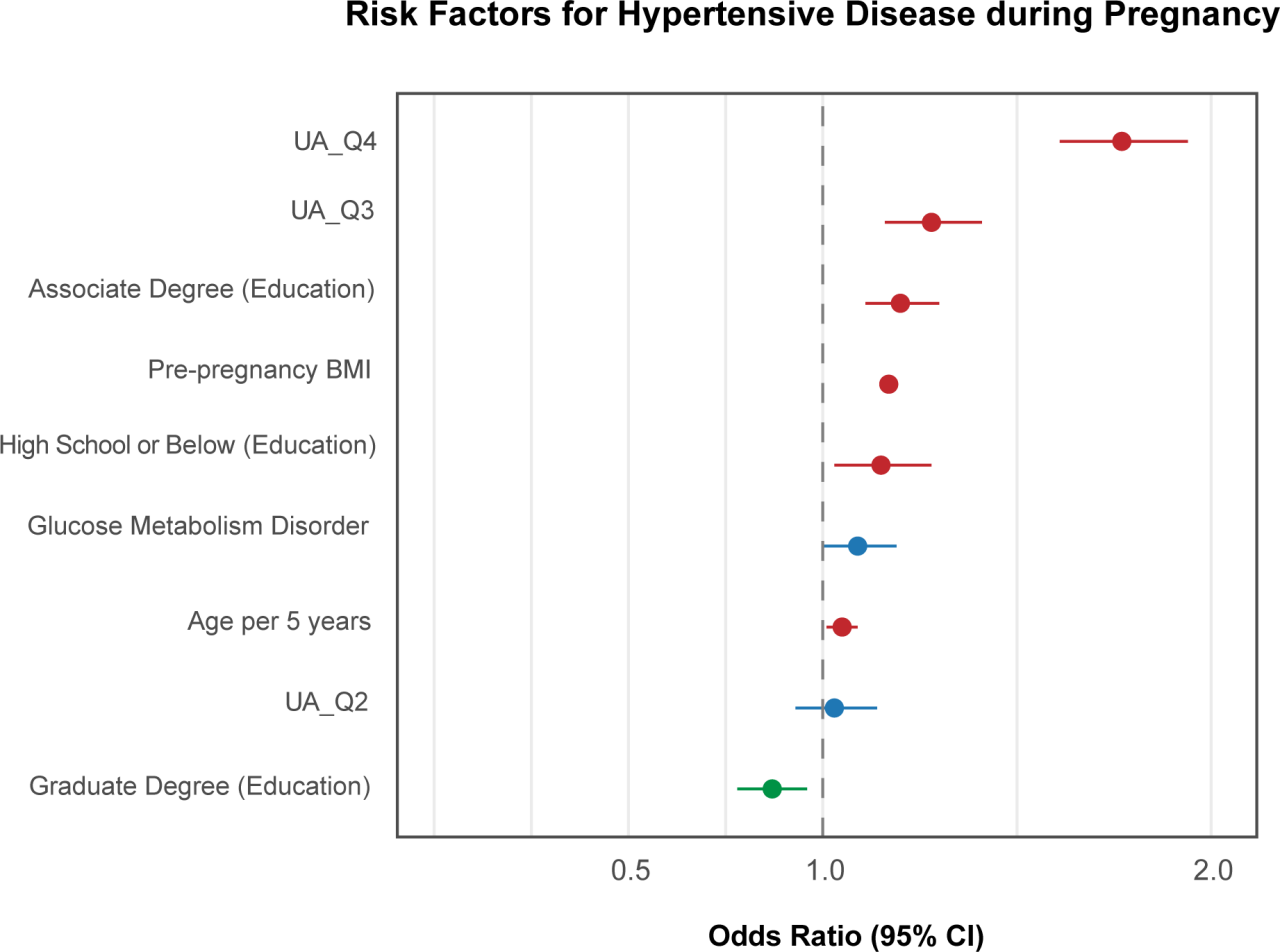
Forest plot of aOR for HDP. This forest plot shows the relationship between UA quartiles and HDP, adjusting for education, parity, BMI, diabetes mellitus, and age per 5-year increase.

In all, the Q3 (in GH and the HDP perspective) and Q4 (in all three types of hypertensive outcomes) quantiles outperformed other maternal covariates, including pre-pregnancy BMI as an effective indicator (**Figures 7**–**9**), which suggests that maternal UA above the average during pregnancy serves as an important feature of HDP development.

### 3.5 Stratified analyses on the association of maternal UA with hypertensive outcomes

**Figure 10:**
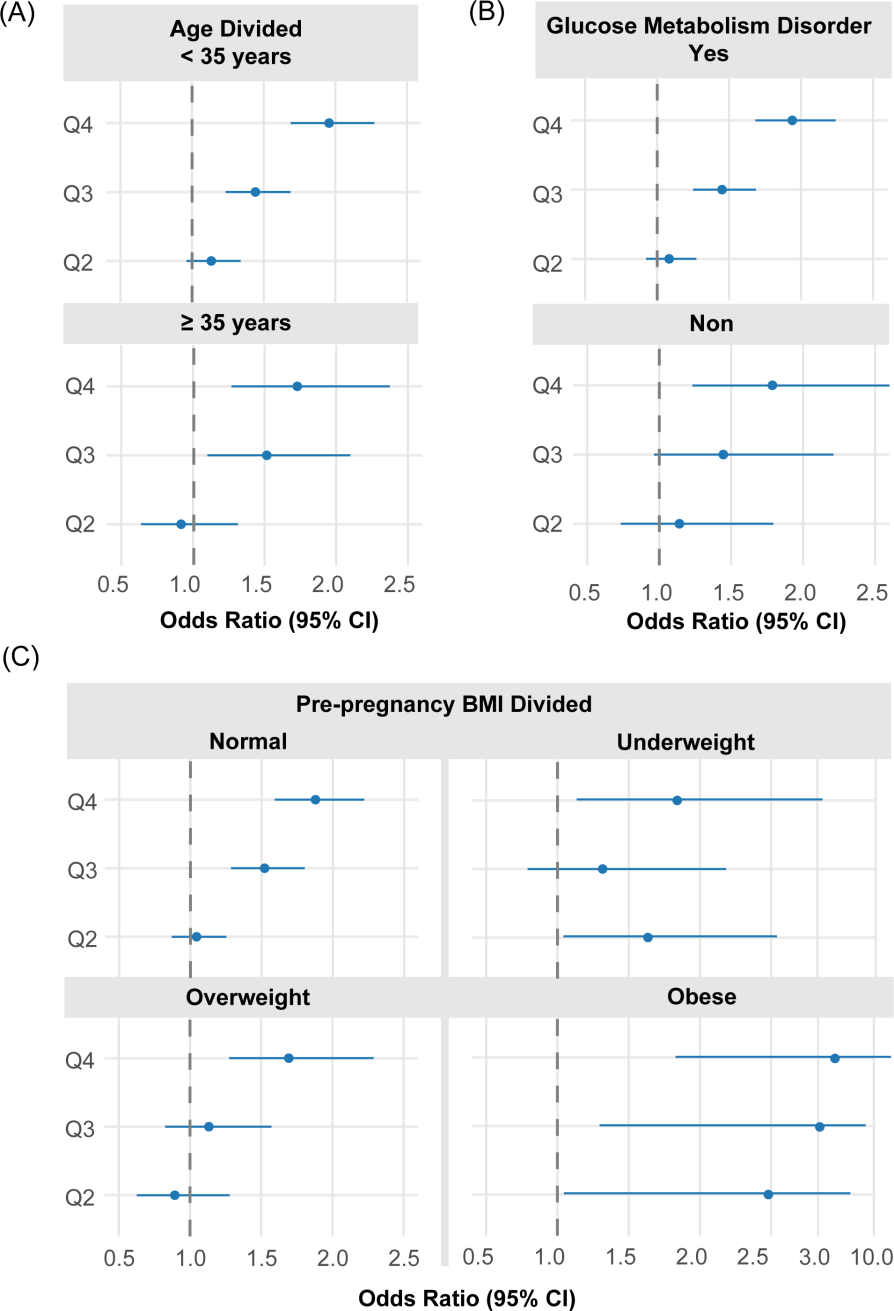
Stratified forest plots of aOR for GH by UA quartiles across key subgroups. **(A)** Stratification by age group; **(B)** Stratification by diabetes status; **(C)** Stratification by BMI category. Each subgroup analysis controlled for parity, BMI (where not the stratifying variable), diabetes, and age.

The stratified analyses depend on demographic and clinical factors (including education levels, pre-pregnancy BMI, conception age, and GMD status) are presented, all used population with UA in Q1 as the reference group. Overall, except in the underpowered subgroup (the underweight divided by pre-pregnancy BMI) due to the limited sample sizes, GH risk (**Table 4**, **Figure 10**) increased consistently across ascending serum UA quartiles (Q2 to Q4), with the highest quartile (Q4) commonly bearing the greatest burden of disease risk. Among pre-pregnancy obese cases, GH risk was markedly accentuated, with the aOR increasing progressively from 2.48 in the Q2 cohort to 3.96 in the Q4 cohort. No substantial difference in GH risk was observed when divided by conception age of 35 years. In the GMD subgroup, GH risk was significantly elevated from Q3 onward (Q3 aOR = 1.45, Q4 aOR = 1.94), whereas in non-GMD participants, significance was reached only in Q4 (aOR = 1.79).

**Table 4:** Stratified associations between maternal serum UA quartiles and GH according to education, pre-pregnancy BMI, age, and glucose metabolism disorders.

			Estimate	Conf. low	Conf. high	SE	P-value
*Pre-Pregnancy BMI*	Normal	*Q2*	1.04	0.87	1.25	0.09	0.652
		*Q3*	1.52	1.29	1.80	0.09	0.000
		*Q4*	1.88	1.59	2.22	0.09	0.000
	Obese	*Q2*	2.48	1.03	6.91	0.48	0.056
		*Q3*	3.09	1.40	8.20	0.44	0.011
		*Q4*	3.96	1.88	10.22	0.43	0.001
	Overweight	*Q2*	0.89	0.63	1.28	0.18	0.534
		*Q3*	1.13	0.83	1.57	0.16	0.447
		*Q4*	1.69	1.28	2.29	0.15	0.000
	Underweight	*Q2*	1.64	1.04	2.61	0.24	0.036
		*Q3*	1.32	0.79	2.18	0.26	0.286
		*Q4*	1.84	1.10	3.06	0.26	0.018
*Age*	< 35 years	*Q2*	1.13	0.96	1.34	0.09	0.138
		*Q3*	1.44	1.24	1.69	0.08	0.000
		*Q4*	1.96	1.69	2.27	0.08	0.000
	≥35 years	*Q2*	0.92	0.64	1.31	0.18	0.640
		*Q3*	1.52	1.10	2.10	0.16	0.011
		*Q4*	1.73	1.27	2.37	0.16	0.001
*GMD Status*	GMD	*Q2*	1.08	0.92	1.27	0.08	0.327
		*Q3*	1.45	1.25	1.69	0.08	0.000
		*Q4*	1.94	1.68	2.24	0.07	0.000
	Non-GMD	*Q2*	1.14	0.73	1.79	0.23	0.565
		*Q3*	1.45	0.96	2.21	0.21	0.081
		*Q4*	1.79	1.23	2.67	0.20	0.003

aOR for GH across serum UA quartiles (Q2–Q4, reference = Q1) are presented separately within pre-pregnancy BMI (normal, obese, overweight, underweight), age (< 35 vs ≥ 35 years), and GMD status. BMI, Body Mass Index; conf, confidence lower bound; GMD, Glucose Metabolism Disorder; SE, Standard Error.

**Figure 11:**
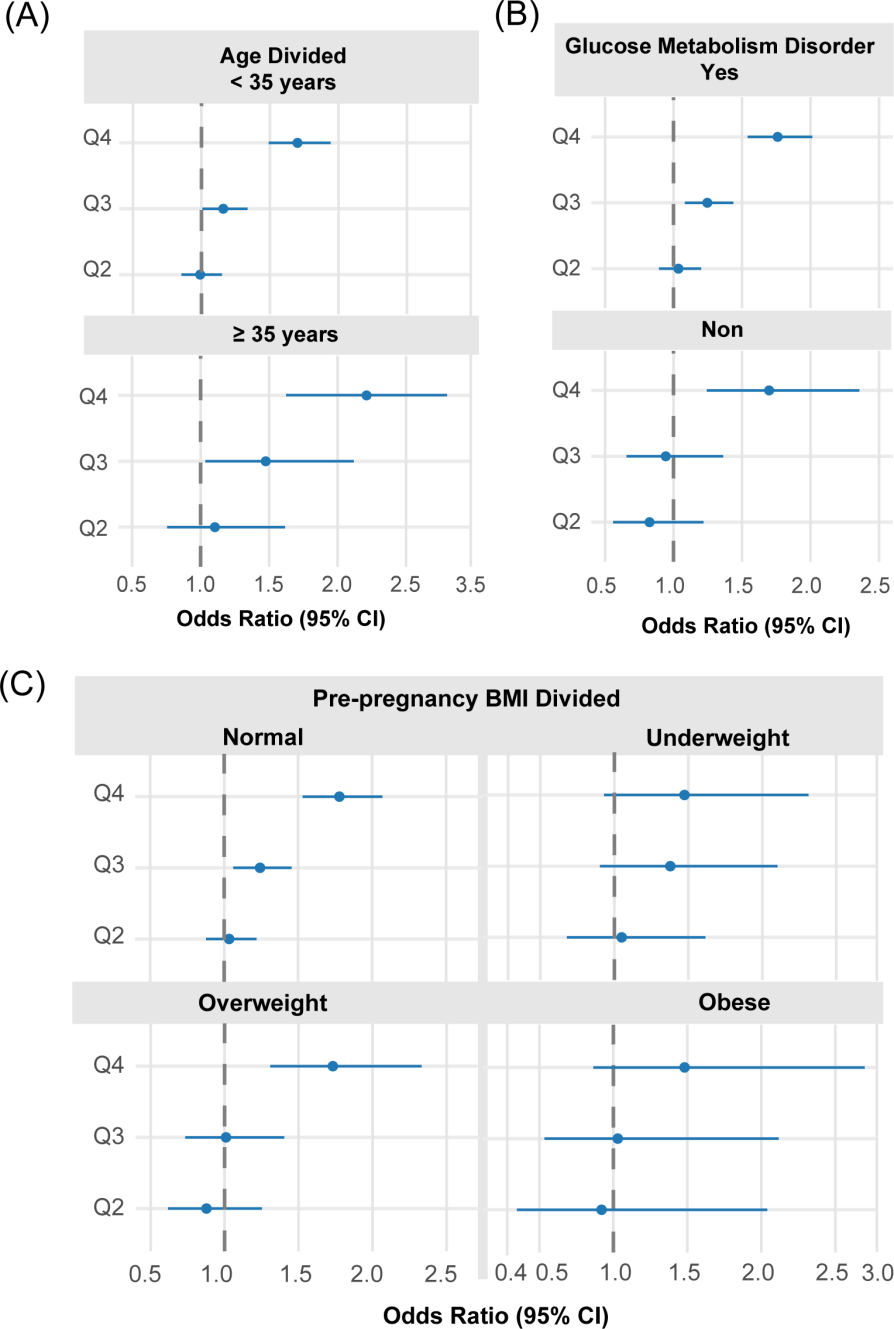
Stratified forest plots of aOR for PE by UA quartiles across key subgroups. **(A)** Stratification by age group; **(B)** Stratification by diabetes status; **(C)** Stratification by BMI category. Each subgroup analysis controlled for parity, BMI (where not the stratifying variable), diabetes, and age.

**Figure 12:**
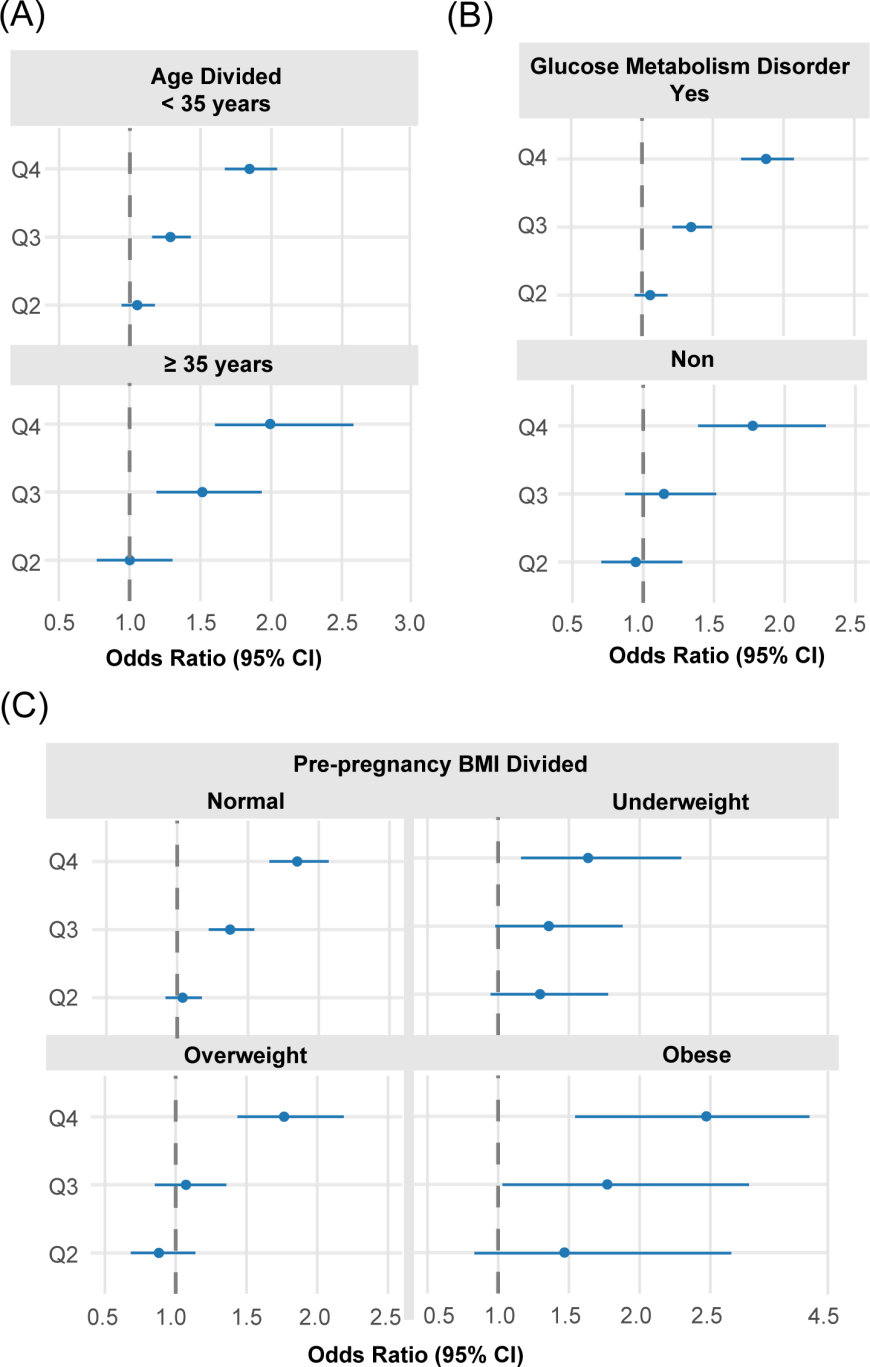
Stratified forest plots of aOR for overall HDP status by UA quartiles across key subgroups. **(A)** Stratification by age group; **(B)** Stratification by diabetes status; **(C)** Stratification by BMI category. Each subgroup analysis controlled for parity, BMI (where not the stratifying variable), diabetes, and age.

Likewise, PE risk (**Table 5**, **Figure 11**) rose progressively across UA level in Q2 to Q4, with the most pronounced elevation observed in Q4. When stratified by pre-pregnancy BMI, PE risk rose significantly in the Q4 cohort among women with normal weight (aOR = 1.78) and those who were overweight (aOR = 1.73); in obese women, no statistically significant association was observed across any quartile. Distinct from GH, the association between serum UA and PE differed by maternal age. Among younger women, elevated UA conferred a significant risk in Q3, Q4, whereas in the advanced conception age group (≥35 years), the relationship was stronger, with Q4 aOR of 2.21 versus 1.70 in the younger group. UA–PE association was more consistent among women with GMD, showing significant elevations from Q3 onward (aOR = 1.25 and 1.76, respectively), whereas in non-GMD women, significance was reached only in Q4 (aOR = 1.70). Combinedly, the effect of maternal UA level differed most significantly when divided by GMD status and pre-pregnancy BMI (**Table 6**, **Figure 12**).

**Table 5:** Stratified associations between maternal serum UA quartiles and PE according to education, pre-pregnancy BMI, age, and glucose metabolism disorders.

			Estimate	Conf. low	Conf. high	SE	P-value
*Pre-Pregnancy BMI*	Normal	*Q2*	1.03	0.88	1.22	0.08	0.693
		*Q3*	1.24	1.06	1.46	0.08	0.007
		*Q4*	1.78	1.53	2.07	0.08	0.000
	Obese	*Q2*	0.92	0.43	2.04	0.40	0.833
		*Q3*	1.03	0.53	2.12	0.35	0.932
		*Q4*	1.48	0.84	2.88	0.31	0.210
	Overweight	*Q2*	0.88	0.62	1.25	0.18	0.468
		*Q3*	1.01	0.73	1.40	0.17	0.953
		*Q4*	1.73	1.31	2.33	0.15	0.000
	Underweight	*Q2*	1.05	0.68	1.62	0.22	0.825
		*Q3*	1.38	0.90	2.10	0.22	0.136
		*Q4*	1.47	0.93	2.31	0.23	0.094
*Age*	< 35 years	*Q2*	0.99	0.85	1.15	0.08	0.905
		*Q3*	1.16	1.01	1.34	0.07	0.040
		*Q4*	1.70	1.49	1.94	0.07	0.000
	≥35 years	*Q2*	1.10	0.76	1.62	0.19	0.607
		*Q3*	1.48	1.04	2.12	0.18	0.033
		*Q4*	2.21	1.59	3.11	0.17	0.000
*GMD Status*	GMD	*Q2*	1.04	0.89	1.20	0.08	0.641
		*Q3*	1.25	1.08	1.44	0.07	0.002
		*Q4*	1.76	1.54	2.01	0.07	0.000
	Non-GMD	*Q2*	0.83	0.56	1.22	0.20	0.332
		*Q3*	0.94	0.66	1.36	0.19	0.753
		*Q4*	1.70	1.24	2.36	0.16	0.001

aOR for PE across serum UA quartiles (Q2–Q4, reference = Q1) are presented separately within pre-pregnancy BMI (normal, obese, overweight, underweight), age (< 35 vs ≥ 35 years), and GMD status. BMI, Body Mass Index; conf, confidence lower bound; GMD, Glucose Metabolism Disorder; SE, Standard Error.

**Table 6:** Stratified associations between maternal serum UA quartiles and HDP according to education, pre-pregnancy BMI, age, and glucose metabolism disorders.

			Estimate	Conf. low	Conf. high	SE	P-value
*Pre-Pregnancy BMI*	Normal	*Q2*	1.04	0.92	1.17	0.06	0.548
		*Q3*	1.37	1.22	1.54	0.06	0.000
		*Q4*	1.85	1.65	2.07	0.06	0.000
	Obese	*Q2*	1.47	0.82	2.73	0.31	0.210
		*Q3*	1.77	1.05	3.13	0.28	0.039
		*Q4*	2.47	1.53	4.23	0.26	0.000
	Overweight	*Q2*	0.88	0.68	1.14	0.13	0.332
		*Q3*	1.07	0.85	1.36	0.12	0.553
		*Q4*	1.77	1.44	2.19	0.11	0.000
	Underweight	*Q2*	1.30	0.95	1.78	0.16	0.106
		*Q3*	1.36	0.98	1.88	0.17	0.065
		*Q4*	1.64	1.16	2.29	0.17	0.005
*Age*	< 35 years	*Q2*	1.05	0.94	1.18	0.06	0.370
		*Q3*	1.29	1.16	1.43	0.05	0.000
		*Q4*	1.85	1.67	2.04	0.05	0.000
	≥35 years	*Q2*	1.00	0.77	1.30	0.13	0.993
		*Q3*	1.51	1.19	1.93	0.12	0.001
		*Q4*	1.99	1.59	2.52	0.12	0.000
*GMD Status*	GMD	*Q2*	1.06	0.95	1.18	0.06	0.316
		*Q3*	1.35	1.22	1.50	0.05	0.000
		*Q4*	1.88	1.70	2.07	0.05	0.000
	Non-GMD	*Q2*	0.95	0.70	1.28	0.15	0.719
		*Q3*	1.15	0.87	1.52	0.14	0.331
		*Q4*	1.77	1.39	2.29	0.13	0.000

aOR for HDP across serum UA quartiles (Q2–Q4, reference = Q1) are presented separately within pre-pregnancy BMI (normal, obese, overweight, underweight), age (< 35 vs ≥ 35 years), and GMD status. BMI, Body Mass Index; conf, confidence lower bound; GMD, Glucose Metabolism Disorder; SE, Standard Error.

## 4 Discussion

The large-scale longitudinal study provided compelling evidence regarding the differential positive associations of maternal serum UA levels with each hypertensive outcome. UA levels were meticulously tracked throughout early to mid-pregnancy; the changing trends along gestational age are similar between the normotensive group and those later developing HDP, but the HDP cohort consistently kept a higher UA concentration across the early to mid-gestation. The highest 25% of UA exhibited the strongest relation with HDP occurrence compared to any other covariate, including high pre-pregnancy BMI, the educated status of the mothers, etc. The impact of maternal UA on HDP risk is partially dependent on the corresponding gestational age of the UA data, and such relations are HDP subtype-specific, with strong differentiation between GH and PE. Concluded from our research, a UA level higher than 50^th^ percentile especially when higher than 75^th^ percentile for gestational age should draw more attention. Serial assessments are valuable for identifying persistently elevated UA across sequential measurements, capturing dynamic risk patterns that single measurements may miss. It suggests that UA may serve as a reliable biomarker for identifying deviations from normal pregnancy progression, potentially aiding in early detection and intervention.

Significant elevation of maternal serum UA in early–to mid-gestation likely represents a pathophysiological deviation, rather than a benign variance of pregnancy. Mechanistic and experimental data indicate multiple UA-mediated pathways that converge on vascular dysfunction in all type of hypertensive diseases (19, 20), including endothelial dysfunction due to the reduced nitric oxide (NO) bioavailability (21, 22), for UA inhibits endothelial NO synthase activity and promoting NO scavenging, leading to impaired vasodilation and increased vascular resistance; intracellular uptake of UA triggers pro-oxidative effects like reactive oxygen species (ROS) generation (23), contributing to endothelial and tissue damage (24); local tissue renin–angiotensin system (RAS) activation has been demonstrated in renal, vascular, and adipose tissues, *in vivo* and *in vitro* studies show that UA upregulates RAS components such as angiotensinogen, angiotensin-converting enzyme (ACE), and angiotensin II type 1 receptor (AT1R), thereby promoting vasoconstriction, inflammation, and vascular remodeling (25); UA stimulates vascular smooth muscle cell (VSMC) proliferation and contributes to arteriolar hyalinosis and glomerular afferent arteriole remodeling, which are key features of chronic hypertension and renal damage (26, 27). Such changes brought by UA not only lead to elevation in systemic blood pressure, but also impair renal microcirculation, further exacerbating hypertensive pathology. However, to date there are no large-scale interventional clinical trials on UA−based interventions has been definitively shown to improve outcomes in HDP diseases, only a promising observational and implementation−type studies exploring salivary uric acid (sUA) as a predictive biomarker (28).

Notably, there are also pregnancy-specific effects of high concentrations of UA on the placenta and trophoblast cells’ function, for elevated UA inhibits trophoblast invasion and promotes oxidative stress in villous cells, leading to impaired spiral artery remodeling and reduced uteroplacental perfusion (29, 30). These alterations compromise placental development and function, contributing to the pathogenesis of HDP. When UA rises during the first or early-second trimester, it likely signals early abnormality in placental development or uteroplacental hemodynamics, such as inadequate trophoblast invasion and defective spiral-artery remodeling (31). This early-warning elevation can predict a short-term surge in PE/GH risk. In established PE, hyperuricemia can be attributable to both renal clearance defect, like reduced GFR and altered tubular handling, and ongoing systemic oxidative/endothelial damage (32). In this way, UA acts simultaneously as a marker of severity and as an active pathogenic contributor. Collectively, these UA-driven processes may amplify systemic maternal endothelial activation and renal microvascular injury. In pregnancy, this manifests as disturbed placentation and the maternal syndrome of HDP.

The gestational-age dependent enhancement of UA’s predictive performance for GH/PE also represents a significant finding from the perspective of the clinic. While early pregnancy UA showed good discrimination, measurements after 20 weeks demonstrated even stronger associations, and this may reflect the accumulating placental damage and systemic inflammation as pregnancy progresses. Moreover, our data suggest that elevated UA in GH patients should prompt intensified surveillance for PE progression; Serial UA measurements improve PE prediction over assessments at single time, the optimal predictive threshold may need gestational-age adjustment, which is also supported by ISSHP guidelines (33) and the clinical guideline of hypertension in pregnancy by the Directorate of Women’s Health, Ministry of Health, Trinidad and Tobago. These insights could lead to more personalized and effective monitoring strategies for high-risk pregnancies.

The strengths of the study included the large, prospectively collected cohort, standardized UA measurements, and advanced statistical modeling that accounted for nonlinear relationships, while certain limitations warrant consideration. Important sources of residual confounding could not be fully excluded. Key determinants of uric acid metabolism, such as dietary purine intake, protein consumption, hydration status, renal function indices (e.g., eGFR), use of UA-influencing medications (e.g., diuretics), and socioeconomic factors beyond educational level were unavailable in our dataset. Most of these factors tend to increase UA levels and may therefore bias the observed associations toward overestimation. In addition, this was a single-center study, which may limit generalizability, and potential assay drift or batch effects across the study period could not be fully assessed. Misclassification of HDP outcomes is also possible because diagnoses were extracted from electronic medical records; such misclassification is expected to be non-differential with respect to early-pregnancy UA, likely biasing results toward the null. Although the temporal ordering of UA measurement and HDP diagnosis reduces concerns about reverse causation, early subclinical renal or vascular dysfunction may already elevate UA levels, so reverse causation cannot be entirely excluded.

In conclusion, our findings position serum UA as a robust, clinically accessible biomarker that shows particular promise for predicting GH occurrence and further development, and risk stratification for the population in early pregnancy. The differential associations of maternal UA with HDP subtypes provide insights into disease pathophysiology while supporting personalized monitoring approaches based on UA trajectories. These results strengthen the evidence base for incorporating UA measurement into routine prenatal care protocols for women at risk of hypertensive complications (high pre-pregnancy BMI, low education, etc.). For UA to be integrated as a clinically usable biomarker for hypertensive disorders of pregnancy, several additional steps are required. Standardized analytical methods and clinically meaningful, gestational-age–specific reference ranges must be established, as our findings demonstrate that serum UA rises substantially from 9 to 24 weeks and that risk discrimination depends on gestational timing. Optimal monitoring schedules, including the most informative gestational window and the value of serial rather than single measurements, need to be defined. Moreover, potential UA-guided preventive strategies, such as lifestyle modification (e.g., dietary optimization and reduction of high-purine intake) and antioxidant-based approaches, warrant systematic evaluation given the biological plausibility linking oxidative stress and endothelial dysfunction to HDP. And formal cost-effectiveness analyses and implementation assessments should be incorporated in future study to determine whether UA-based risk stratification meaningfully improves clinical care pathways and maternal–fetal outcomes.

## 5 Conclusion

Elevated maternal serum UA levels are consistently associated with increased risk of HDP outcomes. Pregnant cases with HDP diagnoses maintained higher UA concentrations across early to mid-gestation compared to normotensive controls. The top quartile of UA showed the strongest predictive value for HDP, exceeding traditional risk factors. The UA-HDP association varied by gestational age and HDP subtype (GH and PE). These findings highlight the potential value of maternal UA for early detection and risk stratification of HDP diseases.

## Data Availability

The raw data supporting the conclusions of this article will be made available by the authors, without undue reservation.

## References

[1] Gestational hypertension and preeclampsia: ACOG practice bulletin, number 222. Obstetrics gynecology. (2020) 135:e237–e60. doi: 10.1097/aog.0000000000003891, PMID: 32443079

[2] Wu P Green M Myers JE . Hypertensive disorders of pregnancy. BMJ (Clinical Res ed). (2023) 381:e071653. doi: 10.1136/bmj-2022-071653, PMID: 37391211

[3] Ngene NC Moodley J . Preventing maternal morbidity and mortality from preeclampsia and eclampsia particularly in low- and middle-income countries. Best Pract Res Clin obstetrics gynaecology. (2024) 94:102473. doi: 10.1016/j.bpobgyn.2024.102473, PMID: 38513504

[4] Khedagi AM Bello NA . Hypertensive disorders of pregnancy. Cardiol clinics. (2021) 39:77–90. doi: 10.1016/j.ccl.2020.09.005, PMID: 33222817 PMC7720658

[5] Dimitriadis E Rolnik DL Zhou W Estrada-Gutierrez G Koga K Francisco RPV . Pre-eclampsia. Nat Rev Dis primers. (2023) 9:8. doi: 10.1038/s41572-023-00417-6, PMID: 36797292

[6] Lane-Cordova AD Khan SS Grobman WA Greenland P Shah SJ . Long-term cardiovascular risks associated with adverse pregnancy outcomes: JACC review topic of the week. J Am Coll Cardiol. (2019) 73:2106–16. doi: 10.1016/j.jacc.2018.12.092, PMID: 31023435

[7] Rosenberg EA Seely EW . Update on preeclampsia and hypertensive disorders of pregnancy. Endocrinol Metab Clinics North America. (2024) 53:377–89. doi: 10.1016/j.ecl.2024.05.012, PMID: 39084814

[8] Zhong J Jiang R Liu N Cai Q Cao Q Du Y . Iron-immune crosstalk at the maternal-fetal interface: emerging mechanisms in the pathogenesis of preeclampsia. Antioxidants (Basel Switzerland). (2025) 14. doi: 10.3390/antiox14070890, PMID: 40722994 PMC12292184

[9] Kuwabara M Fukuuchi T Aoki Y Mizuta E Ouchi M Kurajoh M . Exploring the multifaceted nexus of uric acid and health: A review of recent studies on diverse diseases. Biomolecules. (2023) 13. doi: 10.3390/biom13101519, PMID: 37892201 PMC10604821

[10] Maiuolo J Oppedisano F Gratteri S Muscoli C Mollace V . Regulation of uric acid metabolism and excretion. Int J Cardiol. (2016) 213:8–14. doi: 10.1016/j.ijcard.2015.08.109, PMID: 26316329

[11] Gherghina ME Peride I Tiglis M Neagu TP Niculae A Checherita IA . Uric acid and oxidative stress-relationship with cardiovascular, metabolic, and renal impairment. Int J Mol Sci. (2022) 23. doi: 10.3390/ijms23063188, PMID: 35328614 PMC8949471

[12] Kuwabara M Kanbay M Hisatome I . Uric acid and hypertension because of arterial stiffness. Hypertension (Dallas Tex: 1979). (2018) 72:582–4. doi: 10.1161/hypertensionaha.118.11496, PMID: 29987106

[13] Kuwabara M Kodama T Ae R Kanbay M Andres-Hernando A Borghi C . Update in uric acid, hypertension, and cardiovascular diseases. Hypertension research: Off J Japanese Soc Hypertension. (2023) 46:1714–26. doi: 10.1038/s41440-023-01273-3, PMID: 37072573

[14] Piani F Agnoletti D Baracchi A Scarduelli S Verde C Tossetta G . Serum uric acid to creatinine ratio and risk of preeclampsia and adverse pregnancy outcomes. J hypertension. (2023) 41:1333–8. doi: 10.1097/hjh.0000000000003472, PMID: 37260263 PMC10328517

[15] Kametas NA Nzelu D Nicolaides KH . Chronic hypertension and superimposed preeclampsia: screening and diagnosis. Am J obstetrics gynecology. (2022) 226:S1182–s95. doi: 10.1016/j.ajog.2020.11.029, PMID: 35177217

[16] Yildirim A Altinkaynak K Aksoy H Sahin YN Akcay F . Plasma xanthine oxidase, superoxide dismutase and glutathione peroxidase activities and uric acid levels in severe and mild pre-eclampsia. Cell Biochem Funct. (2004) 22:213–7. doi: 10.1002/cbf.1090, PMID: 15248180

[17] Arias-Sánchez C Pérez-Olmos A Reverte V Hernández I Cuevas S Llinás MT . Uric acid and preeclampsia: pathophysiological interactions and the emerging role of inflammasome activation. Antioxidants (Basel Switzerland). (2025) 14. doi: 10.3390/antiox14080928, PMID: 40867825 PMC12382839

[18] Sudjai D Satho P . Relationship between maternal serum uric acid level and preeclampsia with or without severe features. J obstetrics gynaecology: J Institute Obstetrics Gynaecology. (2022) 42:2704–8. doi: 10.1080/01443615.2022.2099254, PMID: 35866243

[19] Saito Y Tanaka A Node K Kobayashi Y . Uric acid and cardiovascular disease: A clinical review. J Cardiol. (2021) 78:51–7. doi: 10.1016/j.jjcc.2020.12.013, PMID: 33388217

[20] Vareldzis R Perez A Reisin E . Hyperuricemia: an intriguing connection to metabolic syndrome, diabetes, kidney disease, and hypertension. Curr hypertension Rep. (2024) 26:237–45. doi: 10.1007/s11906-024-01295-3, PMID: 38270791

[21] Zoccali C Maio R Mallamaci F Sesti G Perticone F . Uric acid and endothelial dysfunction in essential hypertension. J Am Soc Nephrology: JASN. (2006) 17:1466–71. doi: 10.1681/asn.2005090949, PMID: 16611716

[22] Mazzali M Hughes J Kim YG Jefferson JA Kang DH Gordon KL . Elevated uric acid increases blood pressure in the rat by a novel crystal-independent mechanism. Hypertension (Dallas Tex: 1979). (2001) 38:1101–6. doi: 10.1161/hy1101.092839, PMID: 11711505

[23] Ramirez-Sandoval JC Sanchez-Lozada LG Madero M . Uric acid, vascular stiffness, and chronic kidney disease: is there a link? Blood purification. (2017) 43:189–95. doi: 10.1159/000452726, PMID: 28114139

[24] Sautin YY Johnson RJ . Uric acid: the oxidant-antioxidant paradox. Nucleosides nucleotides Nucleic Acids. (2008) 27:608–19. doi: 10.1080/15257770802138558, PMID: 18600514 PMC2895915

[25] Zhang JX Zhang YP Wu QN Chen B . Uric acid induces oxidative stress via an activation of the renin-angiotensin system in 3T3-L1 adipocytes. Endocrine. (2015) 48:135–42. doi: 10.1007/s12020-014-0239-5, PMID: 24671741

[26] Corry DB Eslami P Yamamoto K Nyby MD Makino H Tuck ML . Uric acid stimulates vascular smooth muscle cell proliferation and oxidative stress via the vascular renin-angiotensin system. J hypertension. (2008) 26:269–75. doi: 10.1097/HJH.0b013e3282f240bf, PMID: 18192841

[27] Mazzali M Kanellis J Han L Feng L Xia YY Chen Q . Hyperuricemia induces a primary renal arteriolopathy in rats by a blood pressure-independent mechanism. Am J Physiol Renal Physiol. (2002) 282:F991–7. doi: 10.1152/ajprenal.00283.2001, PMID: 11997315

[28] Püschl IC Bonde L Reading IC Maguire P Macklon NS Van Rijn BB . Salivary uric acid as a predictive test of preeclampsia, pregnancy-induced hypertension and preterm delivery: A pilot study. Acta obstetricia gynecologica Scandinavica. (2020) 99:1339–45. doi: 10.1111/aogs.13888, PMID: 32350850

[29] Aouache R Biquard L Vaiman D Miralles F . Oxidative stress in preeclampsia and placental diseases. Int J Mol Sci. (2018) 19. doi: 10.3390/ijms19051496, PMID: 29772777 PMC5983711

[30] Bainbridge SA Roberts JM . Uric acid as a pathogenic factor in preeclampsia. Placenta. (2008) 29:S67–72. doi: 10.1016/j.placenta.2007.11.001, PMID: 18093648 PMC3319018

[31] Laughon SK Catov J Powers RW Roberts JM Gandley RE . First trimester uric acid and adverse pregnancy outcomes. Am J hypertension. (2011) 24:489–95. doi: 10.1038/ajh.2010.262, PMID: 21252861 PMC3062659

[32] Kang DH Finch J Nakagawa T Karumanchi SA Kanellis J Granger J . Uric acid, endothelial dysfunction and pre-eclampsia: searching for a pathogenetic link. J hypertension. (2004) 22:229–35. doi: 10.1097/00004872-200402000-00001, PMID: 15076175

[33] Magee LA Brown MA Hall DR Gupte S Hennessy A Karumanchi SA . The 2021 International Society for the Study of Hypertension in Pregnancy classification, diagnosis & management recommendations for international practice. Pregnancy Hypertens. (2022) 27:148–69. doi: 10.1016/j.preghy.2021.09.008, PMID: 35066406

