# Supplementary material for: Association of early and mid-pregnancy maternal serum uric acid with hypertensive disorders of pregnancy
Source: Front Endocrinol (Lausanne). 2025 Dec 12;16:1731576. doi: 10.3389/fendo.2025.1731576 (PMC12740876; doi:10.3389/fendo.2025.1731576)
Supplement: Supplementary file 1 [file DataSheet1.doc]

**Table S1. Abbreviation List.**

| **ACE** | angiotensin-converting enzyme |
| --- | --- |
| **aOR** | Adjusted odds ratio |
| **AT1R** | angiotensin II type 1 receptor |
| **BMI** | body mass index |
| **CV** | coefficient of variation |
| **edf** | effective degrees of freedom |
| **GAM** | Generalized additive model |
| **GH** | gestational hypertension |
| **GMD** | glucose metabolism disorder |
| **HDP** | hypertensive disorders of pregnancy |
| **IGT** | impaired glucose tolerance |
| **LOESS** | locally weighted scatterplot smoothing |
| **NO** | nitric oxide |
| **PE** | preeclampsia |
| **PGDM** | pre-gestational diabetes mellitus |
| **RAS** | renin–angiotensin system |
| **ROS** | reactive oxygen species |
| **UA** | uric acid |
| **VSMC** | vascular smooth muscle cell |


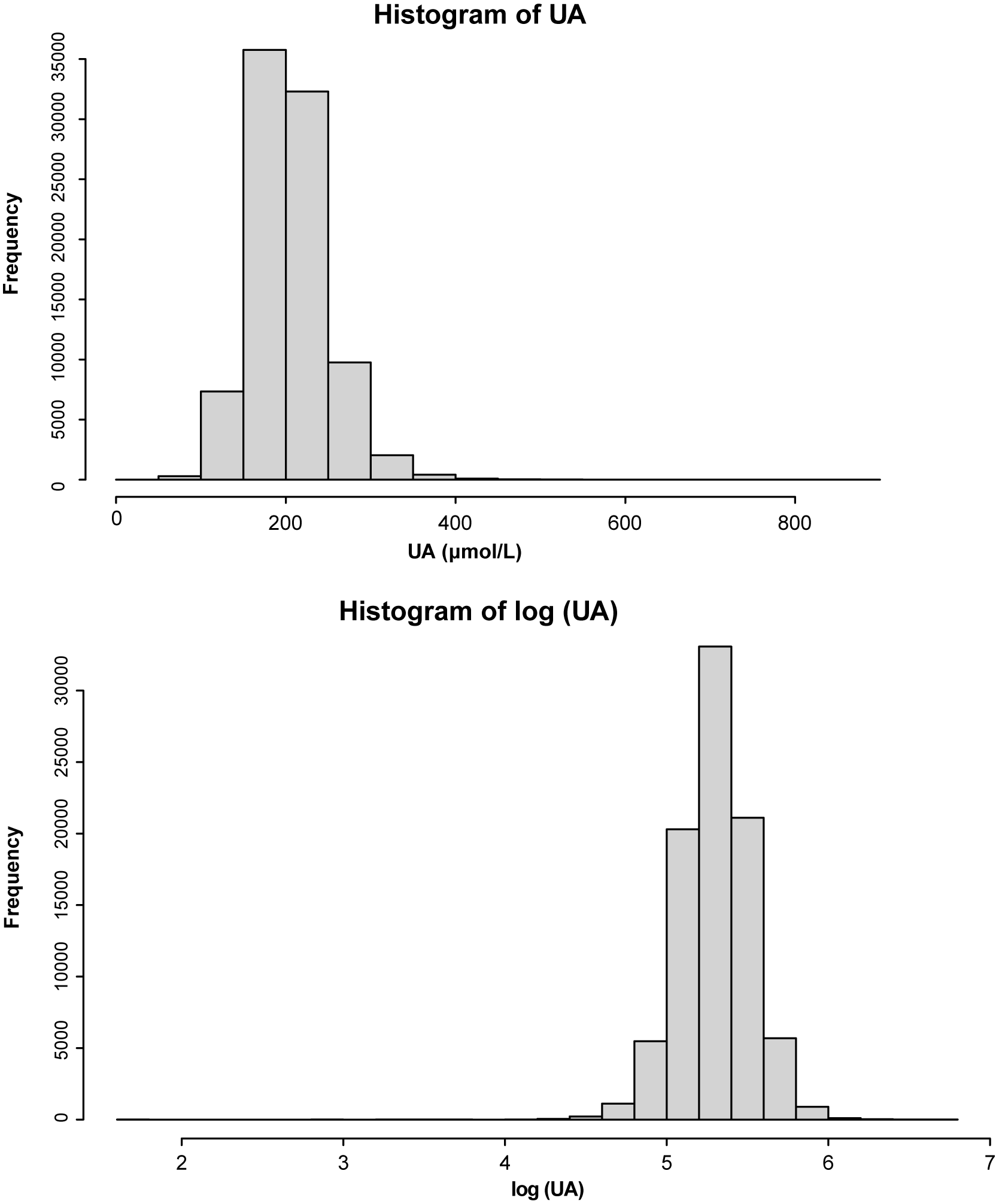


**Supplementary Figure 1.** Frequency distribution of raw maternal serum uric acid (UA, μmol/L, the upper one) and log-transformed UA values (the below one).
